# Prevalence of venous thromboembolism and associated factors in COVID-19 patients at a provincial public hospital in southern Brazil

**DOI:** 10.1590/1677-5449.202401432

**Published:** 2025-08-01

**Authors:** Bruna Valduga Dutra, Luana Valduga Dutra, Henrique Luiz Weber, Matheus Omairi Reinheimer, Matheos Pezzi, Gabriela Teixeira Macalossi, Simone Bonatto, Clandio de Freitas Dutra

**Affiliations:** 1 Universidade de Caxias do Sul – UCS, Caxias do Sul, RS, Brasil.

**Keywords:** COVID-19, venous thrombosis, pulmonary thromboembolism, venous thromboembolism, anticoagulants, excess weight, intubation

## Abstract

**Background:**

COVID-19 is a predominantly respiratory disease, but it also has a strong association with thromboembolism, especially among hospitalized patients.

**Objectives:**

To evaluate the prevalence of venous thromboembolism (VTE) and associated factors in patients with COVID-19 in a public hospital in the interior of South Brazil.

**Methods:**

A retrospective, cross-sectional observational study was carried out by analyzing data from medical records. The VTE outcome was a composite of acute pulmonary thromboembolism (PTE) and deep vein thrombosis (DVT). Associations were analyzed by logistic regression and bivariate analyses using Pearson’s chi-square test.

**Results:**

The sample comprised 964 patients. 56% were male and 44% female, with a mean age of 58.2 ± 15.1 years. 70% of patients were admitted to the ICU, 44.4% died, 97% required oxygen, and 63.7% required intubation. After adjusted analysis, the factors intubation (p=0.02) and prophylactic anticoagulation (p<0.001) were associated with VTE. The following variables were shown to be important risk factors for VTE: intubation (OR 2.3; 95% CI 1.1-4.8, p=0.020) and excess weight (OR 3.3; 95% CI 0.2-2.2, p=0.02), while prophylactic anticoagulation showed a small protective factor (OR 0.02; 95% CI 0.01-0.04, p<0.001).

**Conclusions:**

The results demonstrate how COVID-19, together with several other associated factors, especially intubation, excess weight, and use of anticoagulants, may be related to thromboembolism as risk factors and protective factors. Prophylactic anticoagulation, in particular, was a protective factor.

## INTRODUCTION

The emergence of the SARS-CoV-2 coronavirus in 2019 triggered a global health crisis, causing 700 thousand registered deaths in Brazil up to March of 2023.^1^ The disease, COVID-19, is a syndrome with a wide spectrum of clinical presentations, varying from mild upper respiratory tract disease to a severe respiratory condition needing treatment in an intensive care unit (ICU) and with the potential to cause death.^1^

Its pathophysiology is the result of an exacerbated inflammatory process that tends to occur in a more severe form and with greater frequency among the elderly (over the age of 60 years), people with chronic diseases such as diabetes, hypertension, obesity, kidney disease, or cancer, and patients with cardiac diseases.^2^ Although COVID-19 is recognized as a predominantly respiratory disease, a high frequency of thrombotic events has been documented among patients, especially those in hospital with the severe form of the disease, characterized by a need for treatment in intensive care and ventilatory support.^2^

The predisposition to immunothrombosis is caused by endothelial dysfunction resulting from a systemic inflammatory response to prolonged immobilization of patients, to the constant hypoxemic state, and to hypercoagulability.^2^

It is therefore important to investigate which variables are most often observed and their respective significance to the occurrence of COVID-19-related venous thromboembolism (VTE).

As such, the objective of this study is to assess the prevalence of VTE and associated factors in patients with COVID-19 admitted to a public hospital in a provincial city in Brazil’s southernmost state, Rio Grande do Sul.

## METHODS

This is a cross-sectional, retrospective, observational study based on analysis of data from the medical records of patients with COVID-19 admitted from March 2020 to December 2021 to the Hospital Geral in Caxias do Sul.

The sample size calculation was based on a thromboembolism prevalence of 14.2%, a 95% confidence level, and an acceptable error of 2.5%, resulting in 749 patients. The result was increased by 10% to allow for losses, resulting in a sample size of 833 patients.

The study included patients of both sexes over the age of 18 years and with a positive SARS-CoV-2 test during the data collection period. Patients were excluded if they had spent less than 1 day in the hospital or if they had been admitted for other reasons, even if they had had a positive COVID-19 test, if the viral infection did not have any clinical repercussions.

The VTE outcome was a composite of the pathologies acute pulmonary thromboembolism (PTE, diagnosed with angiotomography) and deep venous thrombosis (DVT, diagnosed with venous Doppler ultrasonography).

The following exposure variables were analyzed: sex (male or female); age, categorized into age brackets; health variables such as smoking, presence or absence of systemic arterial hypertension, diabetes, cancer, chronic kidney disease (CKD) and chronic obstructive pulmonary disease (COPD); anthropometric data (weight and height, to determine excess weight according to body mass index [BMI], defining excess weight as BMI ≥ 25 kg/m^2^); and variables related to COVID-19, such as prophylactic anticoagulation, prior use of antiplatelet drugs, need for oxygen, intubation, ICU admission, and death.

Data were input to an Excel spreadsheet and exported to the Statistical Package for Social Sciences (SPSS Inc, Chicago, IL), version 21.0. Categorical variables were expressed as absolute frequencies and percentages. Bivariate analyses were performed using Pearson’s chi-square test. Associations between independent variables and VTE were analyzed using logistic regression. Variables with p ≤ 0.20 in the crude analysis were included in the adjusted analysis. Variables with p ≤ 0.05 in the adjusted analysis were considered associated with the outcome. This study was submitted to and approved by the Research Ethics Committee at the institution where it was conducted, under decision number 5.307.579. The Strengthening the Reporting of Observational Studies in Epidemiology guidelines were followed.

## RESULTS

**Figure 1:**
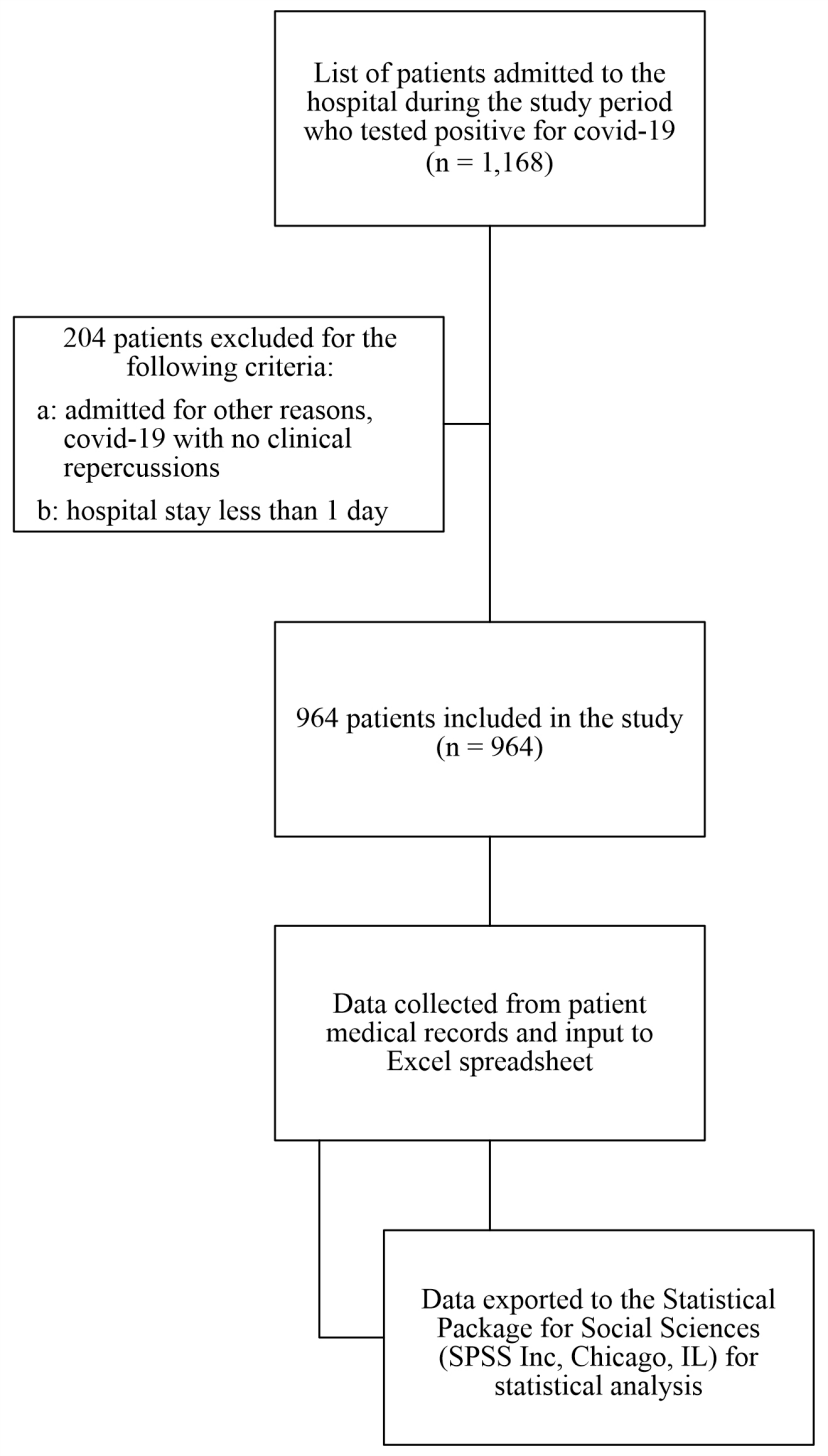
Flowchart illustrating study sample selection.

Initially, a total of 1,168 patients who tested positive for COVID-19 were selected. After application of the exclusion criteria, the final sample comprised 964 patients (Figure 1), 540 (56%) of whom were male and 424 (44%) of whom were female. A majority of these patients were over the age of 60 years (49.1%), with a mean age of 58.2±15.1 years. There was a 22.3% prevalence (215 patients) of smoking (previous or current). Chronic diseases included 31.8% of patients with diabetes, 56.5% with hypertension, 6.7% with COPD, 6.3% with CKD, and 7.3% with a previous diagnosis of cancer. Additionally, 91 patients (9.4%) were taking a platelet aggregation inhibitor prior to admission and 767 (79.6%) were given prophylactic anticoagulation during the hospital stay, which is recommended for all patients admitted to this hospital.

Prophylaxis is institutionalized with enoxaparin 40 mg/day or unfractionated heparin for patients with kidney disease. Of the patients who were not given prophylactic anticoagulation, 146 (74.12%) were on a therapeutic anticoagulation regimen because of some other clinical condition, thromboembolism diagnosed while in hospital, or prior use of anticoagulants. In addition to these, 51 patients (25.88%) were not given any treatment of this type because they were at low risk of VTE and pharmacological prophylaxis was not indicated. Patients given doses exceeding 40 mg de enoxaparin per day, or the equivalent of other drugs, were not defined as being on a prophylactic anticoagulation regime.

Overall, 97.1% of the entire patient sample (n = 964) needed supplemental oxygen, 70.2% were admitted to the ICU, 63.7% needed intubation, and 44.4% died. The sample available for analysis of excess weight was smaller, since only 510 people had complete data for weight and height, with an 83.9% prevalence of excess weight among these COVID-19 patients.

Table 1 lists data on patients diagnosed with PTE (8.9%; 95%CI 7.3-10.9) and the relationships with demographic, anthropometric, health-related, and COVID-19 variables. A total of 274 computed tomography angiographies were performed because of a clinical suspicion of PTE, resulting in 86 positive examinations. The variables intubation (p < 0.001), prophylactic anticoagulation (p < 0.001), prior antiplatelet treatment (p = 0.018), and CKD (p = 0.039) were statistically significant. Of these patients, 83.7% were intubated, revealing a possible association between PTE and a need for intubation, since 63.7% of the entire sample needed intubation. Prophylactic anticoagulation was also associated with PTE (p < 0.001), specifically with a reduced prevalence (11.6% vs. 88.4%). Just 2.3% of the patients who used antiplatelet drugs developed PTE (p = 0.018). CKD was present in 1.2% of these patients. Table 2 describes the relationship between PTE and excess weight, showing that 93% of these patients had excess weight (p = 0.048; n = 510).

**Table 1:** Factors associated with PTE, DVT, and VTE among patients with COVID-19 in a provincial public hospital (n = 964).

**Variable**	**Total (n = 964)**	**PTE (n = 86)**	**p**	**DVT (n = 22)**	**p**	**VTE (n = 105)**	**p**
Sex (%)			0.789		0.565		0.557
Male	540 (56.0)	47 (54.7)		11 (50.0)		56 (53.3)	
Female	424 (44.0)	39 (45.3)		11 (50.0)		49 (46.7)	
Age (%)			0.690*		0.864*		0.759*
< 40 years	122 (12.7)	8 (9.3)		2 (9.1)		10 (9.5)	
40-49 years	148 (15.4)	16 (18.6)		3 (13.6)		19 (18.1)	
50-59 years	221 (22.9)	19 (22.1)		9 (40.9)		25 (23.8)	
≥ 60 years	473 (49.1)	43 (50.0)		8 (36.4)		51 (48.6)	
Smoking (%)			0.824		0.604**		0.521
No	749 (77.7)	66 (76.7)		16 (72.7)		79 (75.2)	
Yes	215 (22.3)	20 (23.3)		6 (27.3)		26 (24.8)	
Needed oxygen (%)			0.314**		0.519**		0.352**
No	28 (2.9)	1 (1.2)		0 (0.0)		1 (1.0)	
Yes	936 (97.1)	85 (98.8)		22 (100.0)		104 (99.0)	
ICU (%)			0.103		0.229		0.020
No	287 (29.8)	19 (22.1)		4 (18.2)		21 (20.0)	
Yes	677 (70.2)	67 (77.9)		18 (81.8)		84 (80.0)	
Intubation (%)			< 0.001		0.074		< 0.001
No	350 (36.3)	14 (16.3)		4 (18.2)		17 (16.2)	
Yes	614 (63.7)	72 (83.7)		18 (81.8)		88 (83.8)	
Death (%)			0.522		0.333		0.263
No	536 (55.6)	45 (52.3)		10 (45.5)		53 (50.5)	
Yes	428 (44.4)	41 (47.7)		12 (54.5)		52 (49.5)	
Prophylactic anticoagulation (%)			< 0.001		< 0.001**		< 0.001
No	197 (20.4)	76 (88.4)		17 (77.3)		90 (85.7)	
Yes	767 (79.6)	10 (11.6)		5 (22.7)		15 (14.3)	
Prior antiplatelet treatment (%)			0.018		0.714		0.015
No	873 (90.6)	84 (97.7)		21 (95.5)		102 (97.1)	
Yes	91 (9.4)	2 (2.3)		1 (4.5)		3 (2.9)	
Arterial hypertension (%)			0.712		0.121		0.582
No	419 (43.5)	39 (45.3)		6 (27,3)		43 (41.0)	
Yes	545 (56.5)	47 (54.7)		16 (72.7)		62 (59.0)	
Diabetes (%)			0.411		0.645		0.750
No	657 (68.2)	62 (72.1)		14 (63.6)		73 (69.5)	
Yes	307 (31.8)	24 (27.9)		8 (36.4)		32 (30.5)	
Cancer (%)			0.588		0.670**		0.804
No	894 (92.7)	81 (94.2)		20 (90.9)		98 (93.3)	
Yes	70 (7.3)	5 (5.8)		2 (9.1)		7 (6.7)	
CKD (%)			0.039		0.645**		0.122
No	903 (93.7)	85 (98.8)		20 (90.9)		102 (97.1)	
Yes	61 (6.3)	1 (1.2)		2 (9.1)		3 (2.9)	
COPD (%)			0.719		0.655**		0.974
No	899 (93.3)	81 (94.2)		20 (90.9)		98 (93.3)	
Yes	65 (6.7)	5 (5.8)		2 (9.1)		7 (6.7)	

* Linear association;

** Fisher’s exact test.

PTE = acute pulmonary thromboembolism; DVT = deep venous thrombosis; VTE = venous thromboembolism; CKD = chronic kidney disease; COPD = chronic obstructive pulmonary disease; ICU = intensive care unit.

**Table 2:** PTE, DVT, and VTE by excess weight among patients with COVID-19 in a provincial public hospital (n = 510).

**Variable**	**Total (n = 510)**	**PTE (n = 57)**	**p**	**DVT (n = 12)**	**p**	**VTE (n = 66)**	**p**
Excess weight (%)			0.048		0.229*		0.018
No	82 (16.1)	4 (7.0)		0 (0.0)		4 (6.1)	
Yes	428 (83.9)	53 (93.0)		12 (100.0)		62 (93.9)	

Excess weight = BMI ≥ 25 kg/m^2^;

* Fisher’s exact test.

PTE = acute pulmonary thromboembolism; DVT = deep venous thrombosis; VTE = venous thromboembolism.

With regard to DVT, a total of 85 Doppler ultrasonography examinations of the lower limbs were performed because of clinical suspicion, confirming 22 cases, equating to a prevalence of 2.3% (95%CI 1.5-3.4). Among this group of patients, the only statistically significant factor was prophylactic anticoagulation (p < 0.001), with 22.7% of patients who had prophylactic anticoagulation developing DVT. These data are shown in Table 1.

The prevalence of thromboembolic events (VTE = PTE + DVT) among patients with COVID-19 was 10.9% (95%CI 9.1-13.0). Of these, 86 patients had a diagnosis of PTE confirmed by angiotomography, 22 had a diagnosis of DVT confirmed by venous Doppler ultrasonography, and three developed concomitant PTE and DVT. The following factors were associated with VTE: ICU (p = 0.020), intubation (p < 0.001), prophylactic anticoagulation (p < 0.001), prior antiplatelet drugs (p = 0.015), and excess weight (data detailed in Tables 1 and 2). Of the 105 patients with VTE, 80% were admitted to the ICU and 83.8% were intubated, demonstrating an important relationship between thromboembolic events and patients with COVID-19 on mechanical ventilation. With regard to use of prophylactic medication and prior antiplatelet treatment, 14.3% and 2.9% of the patients in the VTE group used these medications respectively. In turn, 93.9% of these patients had excess weight.

Table 2 details the association between PTE, DVT, and VTE and excess weight among the patients with COVID-19. Of a total of 510 patients analyzed for this variable, 66 developed VTE, almost 94% of whom had excess weight. Excess weight proved to be a risk factor for VTE and the likelihood of developing the disease was three times greater among overweight patients (OR 3.3; 95%CI 0.2-2.2; p = 0.02) (data not shown in the tables).

Table 3 lists factors associated with VTE in patients with COVID-19, according to the logistic regression analysis. In the crude analysis, factors associated with VTE were admission to the ICU, intubation, use of prophylactic anticoagulation and prior antiplatelet treatment, and CKD. The variables that remained associated with VTE in the adjusted analysis were intubation (p = 0.020) and prophylactic anticoagulation (p < 0.001). Intubation proved to be an important risk factor for VTE, with a 2.3 times greater likelihood of developing the disease among intubated patients (OR 2.3; 95%CI 1.1-4.8; p = 0.020). Prophylactic anticoagulation was a protective factor against the prevalence of VTE (OR 0.02; 95%CI 0.01-0.04; p < 0.001).

**Table 3:** Factors associated with venous thromboembolism among patients with COVID-19 in a provincial public hospital, by logistic regression (n = 964).

**Variable**	**OR (95%CI) crude**	**OR (95%CI) adjusted**	**p**
ICU	1.8 (1.1-2.9)	1.4 (0.7-2.7)	0.396
Intubation	3.3 (1.9-5.6)	2.3 (1.1-4.8)	0.020
Prophylactic anticoagulation	0.02 (0.01-0.04)	0.02 (0.01-0.04)	<0.001
Prior antiplatelet treatment	0.26 (0.08-0.83)	0.32 (0.08-1.20)	0.090
CKD	0.40 (0.1-1.3)	0.31 (0.08-1.13)	0.076

OR = odds ratio; ICU = intensive care unit; CKD = chronic kidney disease.

## DISCUSSION

In this study, we observed an association between prevalence of VTE and the factors intubation and prophylactic anticoagulation. The prevalence of VTE among patients admitted to an ICU appears to be associated with prolonged immobilization and the severity of COVID-19.^3^ In our entire sample, 70.2% of the patients were admitted to the ICU and the prevalence of VTE among these patients was 12.4%. These results are similar to those reported by Tan et al.,^3^ with a VTE prevalence of 14.2%, with a large proportion of patients in the ICU and with potentially more severe presentations. However, in a different study, conducted by Hill et al.,^4^ the prevalence of VTE was less than 10%, even among patients on mechanical ventilation. This difference between prevalence rates may be related to factors such as disease severity, a higher mean patient age, the number of associated comorbidities, a COVID-19 strain that causes a potentially more severe disease, and screening for VTE in both symptomatic and asymptomatic patients.^4^ In a meta-analysis conducted by Di Minno et al.^5^ during the COVID-19 outbreak in Italy at the start of 2020, the prevalence of VTE reached 30%.^5^ In that study, the mean age of patients was 64 years, they were predominantly male, and all were admitted to an ICU. Incidentally, 78% of the patients in that sample were also on prophylactic anticoagulation. The problem with this meta-analysis, however, was the considerable heterogeneity of the studies selected and the way that VTE was defined, introducing several biases. The most important was the number of studies with more patients admitted to ICUs than patients in wards, leading to much higher thromboembolism rates.^5^

Another systematic review, conducted by Brazilian researchers, arrived at similar results, with varied samples of patients in ICUs and wards. This study observed a higher rate of thromboembolic events among patients with severe infections needing intensive care, although part of the sample had VTE associated with oligosymptomatic respiratory infections.^6^

As such, the prevalence rates found in our study are consistent with those found in samples in which most patients were in ICUs and on invasive mechanical ventilation, despite prophylactic anticoagulation.

In all, 80% of the patients with VTE were in intensive care, demonstrating that the severity of COVID-19 and, as a consequence, the need for intensive care, are related to VTE. Similarly, since these are more severe cases and with greater pulmonary involvement, the need for intubation is more frequent, which also explains the association between VTE and intubation (OR 2.3; 95%CI 1.1-4.8; p = 0.020), showing the extent to which invasive mechanical ventilation is associated with VTE, especially when PTE is involved. In the sample analyzed, 44.4% of the patients died, demonstrating that these were more serious cases. Additionally, 614 patients were intubated, 88 of whom exhibited VTE, accounting for 83.8% of the patients with this disease.

In our study, the association between VTE and intubation was similar to the rate observed by Lobbes et al.^7^ in a meta-analysis (OR 2.61; 95%CI 1.94-3.51) in which other factors related to disease severity were also strongly associated as risk factors for VTE, showing how more severe cases cause a powerful association between VTE and intubation.

Prophylactic anticoagulation proved mildly protective against VTE (OR 0.02; 95%CI 0.01-0.04; p < 0.001). More consistent results were observed by Birkeland et al.^8^ (OR 0.58; 95%CI 0.36-0.92; p = 0.02). In their systematic review study, the prevalence of VTE was 26.3%, and they included treatment with anticoagulation at prophylactic doses and therapeutic doses in the analysis, with more than 80% of all patients admitted put on one of these treatment regimens. These samples were selected from patients treated in the wards or in ICUs, with an OR for VTE among patients in ICUs of 6.38 (95%CI 3.67-11.11; *P*<0,001).^8^

In our entire sample, 79.6% of the patients were on a prophylactic anticoagulation regimen and just 2% of these developed VTE. Within the group that did not receive prophylactic anticoagulation, 74.12% of the patients were on anticoagulation at therapeutic doses and the remainder (25.88%) were on anticoagulation for other reasons. Moreover, 80% of the 105 patients who developed VTE were in the ICU, although the factor ICU was not associated with VTE in the adjusted analysis (OR 1,4; IC95% 0,7-2,7, p=0,396). These are possibly some of the reasons why we observed a lower prevalence of VTE when compared to other studies. Another reason for the finding may have been the data collection period, since as the pandemic progressed more and more studies were published demonstrating the importance of prophylactic anticoagulation for all patients hospitalized with COVID-19.

Moreover, it is known that the incidence of thrombotic events related to COVID-19 varies by region, the patient sample, and the method used to screen for VTE.^9^ In another meta-analysis,^10^ which compared VTE events between patients on prophylactic and therapeutic anticoagulation regimens, the prevalence of VTE was 22.7% in hospitalized patients and 30% in patients in an ICU, with an OR of 0.33 (95%CI 0.14-0.75; p = 0.008; I = 0%) in favor of therapeutic anticoagulation.^10^ In this meta-analysis, the inclusion of studies from different parts of the world resulted in heterogeneous samples, in which VTE was only assessed after clinical manifestations.

In a Brazilian meta-analysis^11^ comparing prophylactic vs. therapeutic anticoagulation, while the samples were also heterogeneous, the results appear to indicate a small benefit of therapeutic anticoagulation for reduction of VTE incidence, although without reducing mortality. In contrast with our study, this meta-analysis did not show any association between prophylactic anticoagulation and development of VTE.

As such, the results do confirm the benefits of prophylactic anticoagulation to reduce the risk of VTE, although the protective factor reduces as disease severity increases. Therefore, the more severe the COVID-19 presentation, the more risk factors for VTE the patient tends to develop and the smaller the protective effect of the intervention.^10^ Notwithstanding, in our study, the fact that almost 20% of the sample was on therapeutic anticoagulation or other forms of anticoagulation, especially patients with VTE events, may have impacted the modest protective value found for prophylactic anticoagulation. In turn, prior use of platelet aggregation inhibitors lost its statistical significance in the regression analysis, since the effect of other variables involved in the model was eliminated.

Excess weight was also a factor that contributed to VTE and almost 94% of the patients who developed VTE had excess weight. Patients with this condition had a three times greater likelihood of developing VTE (OR 3.3; 95%CI; 0.2-2.2; p = 0.02). Similar results were found by Wang et al.,^12^ who assessed the relationship between overweight and obesity and prevalence of VTE. They found a strong association with obesity, defined according to BMI, showing that the likelihood of developing VTE was double among patients with grade I obesity I (OR 2.54; 95%CI 1.05-6.14; p = 0.02) and almost four times greater among patients with grade III obesity (OR 3.95; 95%CI 1.40-11.14; p = 0.02). Excess weight, compounded by the endothelial injury caused by SARS-Cov-2, predisposes to thrombosis. The increased body fat provokes an underlying systemic inflammatory state in the patient which, in association with the inflammatory response to COVID-19, tends to increase the probability of developing VTE.

The present study is subject to certain limitations, such as the reverse causality inherent to cross-sectional studies and the fact that the data were collected from patient medical records, which may have been incorrectly filled out and/or have missing data.

## CONCLUSIONS

In this study, associations were observed between VTE and the factors intubation and prophylactic anticoagulation. Intubated patients had twice the rate of VTE than those who were not intubated and prophylactic anticoagulation exhibited a small protective effect against VTE, probably because of the large number of patients who needed admission to the ICU, in addition to the intubation factor itself. Moreover, excess weight was also a factor associated with VTE in this study, although use of platelet antiaggregants did not prove to be associated in the logistic regression. As such, the results observed provide evidence of the extent to which this disease, in conjunction with several other associated factors, can be related to thromboembolism. Prophylactic anticoagulation demonstrated a protective effect, while intubation was a risk factor for occurrence of VTE.
