# Supplementary material for: Prevalence of venous thromboembolism and associated factors in COVID-19 patients at a provincial public hospital in southern Brazil
Source: J Vasc Bras. 2025 Aug 1;24:e20240143. doi: 10.1590/1677-5449.202401432 (PMC12334124; doi:10.1590/1677-5449.202401432)
Supplement: Translation

# Translation: Prevalência de tromboembolismo venoso e fatores associados em pacientes com COVID-19 em um hospital público do interior no Sul do Brasil

**DOI:** 10.1590/1677-5449.202401431

**Authors:** Bruna Valduga Dutra, Luana Valduga Dutra, Henrique Luiz Weber, Matheus Omairi Reinheimer, Matheos Pezzi, Gabriela Teixeira Macalossi, Simone Bonatto, Clandio de Freitas Dutra

**Affiliations:** 1 Universidade de Caxias do Sul – UCS, Caxias do Sul, RS, Brasil.

**Keywords:** COVID-19, trombose venosa, tromboembolismo pulmonar, tromboembolismo venoso, anticoagulantes, excesso de peso, intubação

## Abstract

**Contexto:**

A covid-19 é uma doença predominantemente respiratória, embora tenha forte associação com o tromboembolismo, principalmente entre pacientes hospitalizados.

**Objetivos:**

Avaliar a prevalência de tromboembolismo venoso (TEV) e os fatores associados em pacientes com covid-19 em um hospital público do interior no Sul do Brasil.

**Métodos:**

Estudo transversal realizado por meio de uma análise dos dados documentados em prontuários. O desfecho TEV foi constituído a partir do tromboembolismo pulmonar agudo e da trombose venosa profunda. As associações foram analisadas por regressão logística e a análise bivariada foi realizada pelo teste do qui-quadrado de Pearson.

**Resultados:**

A amostra contou com 964 pacientes, sendo 56% do sexo masculino e 44% do feminino, com idade média de 58,2±15,1 anos. No total, 70% dos pacientes foram admitidos em unidade de terapia intensiva, 44,4% evoluíram para óbito, 97% necessitaram de oxigênio e 63,7% de intubação. Após análise ajustada, estiveram associados ao TEV os fatores intubação (p = 0,02) e anticoagulação profilática (p < 0,001). Demonstraram ser fatores de risco importantes para TEV as variáveis intubação (*odds ratio* [OR] 2,3; IC95% 1,1-4,8; p = 0,020) e excesso de peso (OR 3,3; IC95% 0,2-2,2; p = 0,02), enquanto a anticoagulação profilática mostrou-se como pequeno fator protetor (OR 0,02; IC95% 0,01-0,04; p < 0,001).

**Conclusões:**

Os resultados encontrados demonstraram o quanto a covid-19, em conjunto com diversos outros fatores associados, destacando-se a intubação, excesso de peso e uso de anticoagulantes, pode estar relacionada ao tromboembolismo, tanto como fator de risco quanto de proteção. A anticoagulação profilática, em especial, mostrou-se como fator protetor para o TEV.

## INTRODUÇÃO

O surgimento do coronavírus SARS-CoV-2 em 2019 representou uma crise de saúde global, resultando em 700 mil mortes registradas no Brasil até março de 2023^1^. A covid-19 é uma síndrome com um amplo espectro de apresentações clínicas, variando desde uma doença leve do trato respiratório superior até uma condição respiratória grave que necessita tratamento em unidade de terapia intensiva (UTI), podendo evoluir para óbito^1^.

Sua fisiopatologia é resultante de um processo inflamatório exacerbado, acometendo de forma mais grave e com maior frequência idosos (acima de 60 anos), portadores de doenças crônicas como diabetes, hipertensão, obesidade, doença renal ou câncer, e pacientes com doenças cardíacas^2^. Ainda que seja reconhecida como uma doença predominantemente respiratória, os eventos trombóticos documentados entre pacientes com covid-19 são altos, especialmente entre aqueles internados com a forma grave da doença, caracterizada pela necessidade de tratamento em regime de terapia intensiva e suporte ventilatório^2^.

Essa predisposição à imunotrombose se deve a uma disfunção endotelial resultante de uma resposta inflamatória sistêmica à imobilização prolongada dos pacientes, ao estado de hipoxemia constante e à hipercoagulabilidade^2^.

Nesse sentido, ainda é importante investigar quais são as variáveis mais encontradas e as suas respectivas significâncias quando ocorre o tromboembolismo venoso (TEV) associado à covid-19.

Sendo assim, o objetivo deste trabalho consiste em avaliar a prevalência de TEV e os fatores associados em pacientes com covid-19 em um hospital público do interior do Rio Grande do Sul, Brasil.

## MÉTODOS

Trata-se de um estudo observacional retrospectivo transversal, baseado na análise de dados documentados em prontuários de pacientes internados no Hospital Geral de Caxias do Sul devido à covid-19, no período de março de 2020 a dezembro de 2021.

Para o cálculo do tamanho da amostra foi considerada uma prevalência de 14,2% de tromboembolismo, um nível de confiança de 95% e um erro aceitável de 2,5%, totalizando 749 pacientes. Adicionou-se, ainda, 10% para perdas, resultando em um tamanho de amostra de 833 pacientes.

Foram incluídos no estudo pacientes de ambos os sexos, com idade superior a 18 anos e com teste positivo para SARS-CoV-2 durante o período de coleta de dados. Excluíram-se os pacientes cuja permanência em ambiente hospitalar foi inferior a um dia e aqueles internados por outras causas, mesmo com teste de covid-19 positivo, mas sem repercussão clínica pela infecção viral.

O desfecho TEV foi constituído pelas patologias tromboembolismo pulmonar agudo (TEP, diagnosticado por meio de angiotomografia) e trombose venosa profunda (TVP, diagnosticada por meio de eco-Doppler venoso).

As variáveis de exposição estudadas foram: sexo (masculino e feminino); idade, categorizada em faixas etárias; variáveis de saúde, como tabagismo, presença ou não de hipertensão arterial sistêmica, diabetes, neoplasias, doença renal crônica (DRC) e doença pulmonar obstrutiva crônica (DPOC); antropométricas (peso e altura, para definição do excesso de peso segundo o índice de massa corporal [IMC], considerando excesso de peso um IMC ≥ 25 kg/m^2^); e variáveis relacionadas à covid-19, como uso de anticoagulação profilática, uso prévio de antiagregantes, necessidade de oxigênio, intubação, internação em UTI e óbito.

Os dados foram digitados em planilha do Excel e exportados para o Statistical Package for Social Sciences (SPSS Inc, Chicago, IL), versão 21.0. As variáveis categóricas foram descritas através de frequências absolutas e percentuais. Na análise bivariada, utilizou-se o teste do qui-quadrado de Pearson. As associações entre as variáveis independentes e o TEV foram analisadas por meio da regressão logística. As variáveis com p ≤ 0,20 na análise bruta foram levadas para análise ajustada. Após ajuste, as variáveis com p ≤ 0,05 foram consideradas associadas ao desfecho. O presente estudo foi submetido e aprovado pelo Comitê de Ética em Pesquisa da instituição na qual foi conduzido, por meio do Parecer n° 5.307.579, e foi realizado seguindo as diretrizes Strengthening the Reporting of Observational Studies in Epidemiology.

## RESULTADOS

Inicialmente, foram selecionados 1.168 pacientes com testagem positiva para covid-19. Após a aplicação dos critérios de exclusão, a amostra final foi constituída por 964 pacientes (Figura 1). Destes 964, 540 (56%) eram do sexo masculino e 424 (44%) do sexo feminino. A maioria dos pacientes possuía mais de 60 anos (49,1%), sendo a idade média de 58,2±15,1 anos. Observou-se uma prevalência de 22,3% (215 pacientes) de tabagismo (prévio ou ativo). Entre as doenças crônicas existentes, verificou-se que 31,8% desses pacientes eram diabéticos, 56,5% eram hipertensos, 6,7% possuíam DPOC, 6,3% possuíam DRC e 7,3% possuíam alguma neoplasia diagnosticada previamente. Além disso, 91 pacientes (9,4%) usavam alguma medicação antiagregante plaquetária previamente à internação, e 767 (79,6%) foram submetidos à anticoagulação profilática durante a internação, medida preconizada para todos os pacientes internados no hospital.

**Figura 1:** *Flowchart* da seleção amostral do estudo.

A profilaxia era institucionalizada com regimes de enoxaparina 40 mg/dia ou heparina não fracionada para os pacientes com doenças renais. Entre os pacientes que não foram submetidos à anticoagulação profilática, 146 (74,12%) estavam sob regime de anticoagulação terapêutica devido a outra condição clínica, quadro de tromboembolismo diagnosticado durante a internação ou uso prévio de anticoagulantes. Além disso, 51 pacientes (25,88%) não foram admitidos em nenhum tratamento por apresentarem baixo risco para TEV, não sendo indicada profilaxia farmacológica. Portanto, pacientes que utilizaram doses superiores a 40 mg de enoxaparina ao dia, ou equivalente com outras medicações, não foram considerados como estando em regime de anticoagulação profilática.

Do total de pacientes selecionados (n = 964), 97,1% necessitaram de oxigênio suplementar em algum grau, 70,2% foram admitidos em UTI, 63,7% necessitaram de intubação, e 44,4% evoluíram para óbito. Quando analisado o excesso de peso nesses pacientes, a amostra total diminuiu. Ao todo, 510 pessoas tiveram os dados completos de peso e altura, com uma prevalência de 83,9% de excesso de peso nos pacientes com covid-19.

Na Tabela 1, estão descritos os pacientes portadores de TEP (8,9%; IC95% 7,3-10,9) e sua relação com as variáveis demográficas, antropométricas, de saúde e relacionadas à covid-19. Foram realizadas 274 angiotomografias devido à suspeita clínica de TEP, resultando em 86 exames positivos. Verificou-se que houve significância estatística para as variáveis intubação (p < 0,001), anticoagulação profilática (p < 0,001), antiagregante prévio (p = 0,018) e DRC (p = 0,039). Desses pacientes, 83,7% foram intubados, apontando uma possível associação entre o TEP e a necessidade de intubação, já que, do total da amostra, 63,7% precisaram de intubação. A anticoagulação profilática também se associou com o TEP (p < 0,001), principalmente na diminuição da sua prevalência (11,6% *versus* 88,4%). Entre os pacientes que fizeram uso do antiagregante plaquetário, apenas 2,3% desenvolveram TEP (p = 0,018). A DRC esteve presente em 1,2% desses pacientes. Na Tabela 2, está descrita a relação entre TEP e excesso de peso, sendo que 93% desses pacientes possuíam excesso de peso (p = 0,048; n = 510).

**Tabela 1:** Fatores associados ao TEP, TVP e TEV em pacientes internados com covid-19 em um hospital público do interior (n = 964).

**Variável**	**Total (n = 964)**	**TEP (n = 86)**	**Valor de p**	**TVP (n = 22)**	**Valor de p**	**TEV (n = 105)**	**Valor de p**
Sexo (%)			0,789		0,565		0,557
Masculino	540 (56,0)	47 (54,7)		11 (50,0)		56 (53,3)	
Feminino	424 (44,0)	39 (45,3)		11 (50,0)		49 (46,7)	
Idade (%)			0,690*		0,864*		0,759*
< 40 anos	122 (12,7)	8 (9,3)		2 (9,1)		10 (9,5)	
40-49 anos	148 (15,4)	16 (18,6)		3 (13,6)		19 (18,1)	
50-59 anos	221 (22,9)	19 (22,1)		9 (40,9)		25 (23,8)	
≥ 60 anos	473 (49,1)	43 (50,0)		8 (36,4)		51 (48,6)	
Tabagismo (%)			0,824		0,604**		0,521
Não	749 (77,7)	66 (76,7)		16 (72,7)		79 (75,2)	
Sim	215 (22,3)	20 (23,3)		6 (27,3)		26 (24,8)	
Necessidade de oxigênio (%)			0,314**		0,519**		0,352**
Não	28 (2,9)	1 (1,2)		0 (0,0)		1 (1,0)	
Sim	936 (97,1)	85 (98,8)		22 (100,0)		104 (99,0)	
UTI (%)			0,103		0,229		0,020
Não	287 (29,8)	19 (22,1)		4 (18,2)		21 (20,0)	
Sim	677 (70,2)	67 (77,9)		18 (81,8)		84 (80,0)	
Intubação (%)			< 0,001		0,074		< 0,001
Não	350 (36,3)	14 (16,3)		4 (18,2)		17 (16,2)	
Sim	614 (63,7)	72 (83,7)		18 (81,8)		88 (83,8)	
Óbito (%)			0,522		0,333		0,263
Não	536 (55,6)	45 (52,3)		10 (45,5)		53 (50,5)	
Sim	428 (44,4)	41 (47,7)		12 (54,5)		52 (49,5)	
Anticoagulação profilática (%)			< 0,001		< 0,001**		< 0,001
Não	197 (20,4)	76 (88,4)		17 (77,3)		90 (85,7)	
Sim	767 (79,6)	10 (11,6)		5 (22,7)		15 (14,3)	
Antiagregante prévio (%)			0,018		0,714		0,015
Não	873 (90,6)	84 (97,7)		21 (95,5)		102 (97,1)	
Sim	91 (9,4)	2 (2,3)		1 (4,5)		3 (2,9)	
Hipertensão arterial (%)			0,712		0,121		0,582
Não	419 (43,5)	39 (45,3)		6 (27.3)		43 (41,0)	
Sim	545 (56,5)	47 (54,7)		16 (72,7)		62 (59,0)	
Diabetes (%)			0,411		0,645		0,750
Não	657 (68,2)	62 (72,1)		14 (63,6)		73 (69,5)	
Sim	307 (31,8)	24 (27,9)		8 (36,4)		32 (30,5)	
Neoplasias (%)			0,588		0,670**		0,804
Não	894 (92,7)	81 (94,2)		20 (90,9)		98 (93,3)	
Sim	70 (7,3)	5 (5,8)		2 (9,1)		7 (6,7)	
DRC (%)			0,039		0,645**		0,122
Não	903 (93,7)	85 (98,8)		20 (90,9)		102 (97,1)	
Sim	61 (6,3)	1 (1,2)		2 (9,1)		3 (2,9)	
DPOC (%)			0,719		0,655**		0,974
Não	899 (93,3)	81 (94,2)		20 (90,9)		98 (93,3)	
Sim	65 (6,7)	5 (5,8)		2 (9,1)		7 (6,7)	

* Associação linear;

** Teste exato de Fisher.

TEP = tromboembolismo pulmonar agudo; TVP = trombose venosa profunda; TEV = tromboembolismo venoso; DRC = doença renal crônica; DPOC = doença pulmonar obstrutiva crônica; UTI = unidade de terapia intensiva.

**Tabela 2:** TEP, TVP e TEV segundo excesso de peso em pacientes internados com covid-19 em um hospital público do interior (n = 510).

**Variável**	**Total (n = 510)**	**TEP (n = 57)**	**Valor de p**	**TVP (n = 12)**	**Valor de p**	**TEV (n = 66)**	**Valor de p**
Excesso de peso (%)			0,048		0,229*		0,018
Não	82 (16,1)	4 (7,0)		0 (0,0)		4 (6,1)	
Sim	428 (83,9)	53 (93,0)		12 (100,0)		62 (93,9)	

Excesso de peso = IMC ≥ 25 kg/m^2^;

* Teste exato de Fisher.

TEP = tromboembolismo pulmonar agudo; TVP = trombose venosa profunda; TEV = tromboembolismo venoso.

No que tange à TVP, foram realizados 85 exames de eco-Doppler de membros inferiores devido à suspeita clínica, dos quais 22 casos foram confirmados, resultando em uma prevalência de 2,3% (IC95% 1,5-3,4). Nesse grupo de pacientes, somente o fator anticoagulação profilática demonstrou significância estatística (p < 0,001), sendo que 22,7% dos pacientes que fizeram seu uso desenvolveram TVP. Os dados estão apresentados na Tabela 1.

A prevalência de eventos tromboembólicos (TEV = TEP + TVP) nos portadores de covid-19 foi de 10,9% (IC95% 9,1-13,0). Destes, 86 pacientes tiveram diagnóstico de TEP confirmado por angiotomografia, 22 tiveram diagnóstico de TVP confirmado por eco-Doppler venoso, e três evoluíram com TEP e TVP concomitante. Estiveram associados ao TEV os seguintes fatores: UTI (p = 0,020), intubação (p < 0,001), anticoagulação profilática (p < 0,001), antiagregante plaquetário prévio (p = 0,015) e excesso de peso (dados descritos nas Tabelas 1 e 2). Dos 105 pacientes com TEV, 80% foram internados em UTI e 83,8% foram intubados, demonstrando uma importante relação entre eventos tromboembólicos e pacientes com covid-19 em ventilação mecânica. Com relação ao uso de medicações profiláticas e prévias, 14,3% e 2,9% dos pacientes do grupo TEV faziam uso dessas medicações, respectivamente. O excesso de peso esteve presente em 93,9% desses pacientes.

Na Tabela 2, está descrita a associação entre TEP, TVP e TEV com excesso de peso em pacientes com covid-19. Do total de 510 pacientes avaliados, 66 desenvolveram TEV, sendo que, entre estes, quase 94% possuíam excesso de peso. O excesso de peso demonstrou ser um fator de risco para TEV, sendo que a chance de desenvolver a doença foi três vezes maior nos pacientes com essa condição (OR 3,3; IC95% 0,2-2,2; p = 0,02) (dados não demonstrados em tabelas).

Na Tabela 3, estão descritos os fatores associados ao TEV em pacientes com covid-19, segundo regressão logística. Na análise bruta, estiveram associados com o TEV a internação em UTI, a intubação, o uso de anticoagulação profilática e antiagregação prévia, e ser portador de DRC. Após a análise ajustada, permaneceram associadas ao TEV as variáveis intubação (p = 0,020) e anticoagulação profilática (p < 0,001). A intubação demonstrou ser um fator de risco importante para TEV, sendo que a chance de desenvolver a doença foi 2,3 vezes maior nos pacientes intubados (OR 2,3; IC95% 1,1-4,8; p = 0,020). A anticoagulação profilática demonstrou ser um fator protetor para a prevalência de TEV (OR 0,02; IC95% 0,01-0,04; p < 0,001).

**Tabela 3:** Fatores associados ao tromboembolismo venoso em pacientes internados com covid-19 em um hospital público do interior, segundo regressão logística (n = 964).

**Variável**	**OR (IC95%) bruta**	**OR (IC95%) ajustada**	**Valor de p**
UTI	1,8 (1,1-2,9)	1,4 (0,7-2,7)	0,396
Intubação	3,3 (1,9-5,6)	2,3 (1,1-4,8)	0,020
Anticoagulação profilática	0,02 (0,01-0,04)	0,02 (0,01-0,04)	<0,001
Antiagregante prévio	0,26 (0,08-0,83)	0,32 (0,08-1,20)	0,090
DRC	0,40 (0,1-1,3)	0,31 (0,08-1,13)	0,076

OR = *odds ratio*; UTI = unidade de terapia intensiva; DRC = doença renal crônica.

## DISCUSSÃO

Neste estudo, observamos uma associação entre a prevalência de TEV e os fatores intubação e anticoagulação profilática. A prevalência de TEV em pacientes admitidos em UTI parece estar associada ao tempo prolongado de imobilização e à gravidade da covid-19^3^. Da amostra total, 70,2% dos pacientes foram admitidos em UTI e, entre estes, a prevalência de TEV foi de 12,4%. Esses resultados são semelhantes aos encontrados por Tan et al.^3^, com prevalência de TEV de 14,2%, sendo que grande parte dos pacientes também estavam em regime de UTI e com quadros potencialmente mais graves. Entretanto, em outro estudo, conduzido por Hill et al.^4^, a prevalência de TEV encontrada foi inferior a 10%, mesmo nos pacientes em ventilação mecânica. Essa diferença entre as prevalências pode estar associada a fatores como a gravidade da doença, a idade média mais alta dos pacientes, a quantidade de comorbidades associadas, a cepa de covid-19 causadora de quadro potencialmente mais grave, e o rastreio para TEV em pacientes sintomáticos e assintomáticos^4^. Em uma metanálise conduzida por Di Minno et al.^5^ durante o surto de covid-19 na Itália, no início do ano de 2020, a prevalência de TEV chegou a 30%. Nesse estudo, a idade média dos pacientes era de 64 anos, sendo predominantemente do sexo masculino, e todos estavam em regime de UTI. Ademais, até 78% dos pacientes dessa amostra estavam, também, sob regime de anticoagulação profilática. A problemática dessa metanálise, contudo, foi a grande heterogeneidade dos estudos selecionados e a forma com que o TEV era definido, gerando diversos vieses. Entre eles, o principal era a quantidade de estudos que englobava mais pacientes internados em UTI do que pacientes em enfermaria, resultando em taxas de tromboembolismo muito mais elevadas^5^.

Uma outra revisão sistemática, realizada por autores brasileiros, chegou a resultados semelhantes, com amostras variadas de pacientes em regime de UTI e enfermaria. Nesse estudo, houve um aumento na taxa de eventos tromboembólicos em pacientes com quadro de infecção grave necessitando de terapia intensiva, embora parte da amostra tenha manifestado TEV em quadros de infecção respiratória oligossintomáticos^6^.

Assim, as prevalências encontradas em nosso estudo são consistentes com aquelas encontradas em amostras que incluem maior parte dos pacientes em regime de UTI e ventilação mecânica invasiva, apesar da anticoagulação profilática.

Do total de pacientes que apresentaram TEV, 80% estavam em ambiente de terapia intensiva, demonstrando que a gravidade da covid-19 e, por conseguinte, a necessidade de terapia intensiva, relacionou-se com o TEV. Da mesma forma, por serem quadros mais graves e com maior acometimento pulmonar, a necessidade de intubação se faz mais presente, o que também explica a associação entre TEV e intubação (OR 2,3; IC95% 1,1-4,8; p = 0,020), evidenciando o quanto a ventilação mecânica invasiva está associada ao TEV, especialmente quando relacionada ao TEP. Na amostra analisada, até 44,4% dos pacientes evoluíram para óbito, demonstrando que se tratava de casos mais graves. Além disso, havia 614 pacientes intubados, dos quais 88 apresentaram TEV, chegando a representar 83,8% dos pacientes com a doença.

Em nosso estudo, a associação entre TEV e intubação foi semelhante à encontrada por Lobbes et al.^7^ em sua metanálise (OR 2,61; IC95% 1,94-3,51), na qual outros fatores relacionados à gravidade da doença também estiveram fortemente associados como fatores de risco para TEV, evidenciando como quadros mais graves geram associação importante entre TEV e intubação.

A anticoagulação profilática demonstrou uma pequena proteção no TEV (OR 0,02; IC95% 0,01-0,04; p < 0,001). Resultados mais consistentes foram encontrados por Birkeland et al.^8^ (OR 0,58; IC95% 0,36-0,92; p = 0,02). Nesse estudo, que se tratava de uma revisão sistemática, a prevalência de TEV foi de 26,3%, sendo incluído tratamento com anticoagulação com doses profiláticas e doses terapêuticas, com mais de 80% dos pacientes admitidos em um desses regimes de tratamento. As amostras foram obtidas de pacientes sendo tratados a nível de enfermaria e UTI, com OR de TEV para pacientes em UTI de 6,38 (IC95% 3,67-11,11; *p<*0,001)^8^.

Em nossa amostra total, 79,6% dos pacientes estavam em regime de anticoagulação profilática e, entre estes, apenas cerca de 2% evoluíram com TEV. Entre esse grupo, que não foi submetido à anticoagulação profilática, 74,12% dos pacientes estavam sob anticoagulação em doses terapêuticas, e o restante (25,88%) estava sob anticoagulação por outras razões. Ademais, dos 105 pacientes que apresentaram TEV, 80% deles estavam em regime de UTI, embora o fator UTI não tenha sido associado ao TEV na análise ajustada (OR 1,4; IC95% 0,7-2,7, p=0,396). Por esse motivo, essas são, possivelmente, algumas das razões pelas quais encontramos uma menor prevalência de TEV quando comparado a outros estudos. Outro motivo para tal achado pode ser o período de coleta de dados, já que, com o avançar da pandemia, cada vez mais estudos foram sendo publicados, evidenciando a importância da anticoagulação profilática para todos os pacientes hospitalizados com covid-19.

Além disso, é sabido que a incidência de eventos trombóticos relacionados à covid-19 varia conforme a região, a amostra de pacientes e o método para *screening* de TEV^9^. Em outra metanálise^10^, na qual se comparou eventos de TEV entre pacientes em regime de anticoagulação profilática e terapêutica, a prevalência de TEV foi de 22,7% em pacientes hospitalizados e 30% em pacientes de UTI, com OR de 0,33 (IC95% 0,14-0,75; p = 0,008; I = 0%), que favoreceu a anticoagulação terapêutica^10^. Nessa metanálise, a presença de estudos de diferentes partes do mundo possibilitou heterogeneidade nas amostras, as quais avaliavam o TEV somente a partir de manifestações clínicas.

Em uma metanálise brasileira^11^, na qual foi comparada a anticoagulação profilática *versus* terapêutica, os resultados encontrados, embora também com heterogeneidade nas amostras, pareceram indicar um pequeno benefício para a anticoagulação terapêutica na redução da incidência de TEV, porém sem reduzir mortalidade. Em contraste com o nosso estudo, essa metanálise não mostrou associação entre a anticoagulação profilática e o desenvolvimento de TEV.

Portanto, os resultados corroboram, de fato, o benefício da anticoagulação profilática para diminuir o risco de TEV, embora o fator protetor diminua conforme aumenta a gravidade da doença. Dessa forma, quanto mais grave for o quadro de covid-19, mais fatores de risco para TEV o paciente tende a desenvolver e menor é o efeito protetor dessa intervenção^10^. Contudo, em nosso estudo, o fato de quase 20% da amostra estar sob regimes de anticoagulação terapêutica ou outras formas de anticoagulação, especialmente os pacientes com eventos de TEV, pode ter resultado no modesto valor protetor encontrado para a anticoagulação profilática. Já o uso prévio de medicações de antiagregação plaquetária perdeu a significância estatística na análise da regressão, uma vez que foi retirado o efeito das outras variáveis envolvidas no modelo.

O excesso de peso também foi um fator que contribuiu para o TEV, sendo que quase 94% dos pacientes que desenvolveram o TEV possuíam excesso de peso. Pacientes com essa condição tiveram três vezes mais chance de desenvolver TEV (OR 3,3; IC95%; 0,2-2,2; p = 0,02). Resultados semelhantes foram encontrados por Wang et al.^12^, que avaliaram a relação entre sobrepeso e obesidade com a prevalência de TEV. Nesse estudo, foi encontrada uma forte associação com a obesidade, definida por meio do IMC, sendo que a chance de desenvolver TEV foi duas vezes maior nos pacientes com obesidade grau I (OR 2,54; IC95% 1,05-6,14; p = 0,02) e quase quatro vezes maior em pacientes com obesidade grau III (OR 3,95; IC95% 1,40-11,14; p = 0,02). O excesso de peso, aliado ao dano endotelial ocasionado pelo SARS-Cov-2, predispõe à trombose. O aumento da gordura corporal leva a um estado inflamatório sistêmico de base no indivíduo que, associado à resposta inflamatória à covid-19, tende ao aumento da probabilidade do desenvolvimento do TEV.

O presente estudo apresenta algumas limitações, como a causalidade reversa, inerente aos estudos transversais, e o fato de os dados terem sido coletados em prontuários, cujos dados podem ter sido inadequadamente preenchidos e/ou estarem faltantes.

## CONCLUSÃO

Neste estudo, observou-se associação do TEV com os fatores intubação e anticoagulação profilática. Pacientes intubados tiveram duas vezes mais TEV do que os que não estavam intubados, e a anticoagulação profilática demonstrou uma pequena proteção ao TEV, provavelmente devido à grande quantidade de pacientes que necessitaram de internação em UTI, além do próprio fator intubação. Além disso, o excesso de peso também foi um fator que se associou ao TEV neste estudo, enquanto o uso de antiagregantes plaquetários não mostrou associação na regressão logística. Neste sentido, os resultados encontrados fornecem subsídios do quanto essa doença, em conjunto com diversos outros fatores associados, pode estar relacionada ao tromboembolismo. A anticoagulação profilática demonstrou fator protetor, enquanto a intubação mostrou-se como fator de risco para ocorrência de TEV.
